# The Dynamic Consequences of Cooperation and Competition in Small-World Networks

**DOI:** 10.1371/journal.pone.0126234

**Published:** 2015-04-30

**Authors:** Iván Y. Fernández-Rosales, Larry S. Liebovitch, Lev Guzmán-Vargas

**Affiliations:** 1 Departamento de Física, Escuela Superior de Física y Matemáticas, Instituto Politécnico Nacional, México DF, México; 2 Department of Physics, Queens College, City University of New York, Flushing, NY, USA; 3 Adjunct Senior Research Scholar, Advanced Consortium on Cooperation, Conflict, and Complexity (AC4), Earth Institute, Columbia University, New York, NY, USA; 4 Unidad Profesional interdisciplinaria en Ingeniería y Tecnologías Avanzadas, Instituto Politécnico Nacional, México DF, México; Wake Forest School of Medicine, UNITED STATES

## Abstract

We present a study of the social dynamics among cooperative and competitive actors interacting on a complex network that has a small-world topology. In this model, the state of each actor depends on its previous state in time, its inertia to change, and the influence of its neighboring actors. Using numerical simulations, we determine how the distribution of final states of the actors and measures of the distances between the values of the actors at local and global levels, depend on the number of cooperative to competitive actors and the connectivity of the actors in the network. We find that similar numbers of cooperative and competitive actors yield the lowest values for the local and global measures of the distances between the values of the actors. On the other hand, when the number of either cooperative or competitive actors dominate the system, then the divergence is largest between the values of the actors. Our findings make new testable predictions on how the dynamics of a conflict depends on the strategies chosen by groups of actors and also have implications for the evolution of behaviors.

## Introduction

In recent years, a number of studies from the perspective of natural sciences [1–5] have sought to understand the complexity of social dynamics. One major question has been to understand the roles played by cooperation and competition. As pointed out by Deutsch [4, 6], these two basic forms of interaction can be identified when people are involved in a given situation. In the simplest terms, a cooperative interaction between two people occurs when their goals are positively correlated, while a competitive interaction happens when their goals are negatively correlated. In many real situations there is a mixture of actors each with their own cooperative or competitive interactions which determines the dynamics of their interaction and their final situation [4, 6–9]. In the context of evolutionary biology and sociobiology, a number of authors have studied cooperation. Nowak suggests that there are five mechanisms involved: kin selection, direct reciprocity, indirect reciprocity, network reciprocity and group selection [10]. Johnson et al. [11] have pointed out that cooperative behavior between groups of individuals is beneficial for improving group productivity, favorable interpersonal relations, and better psychological health. Tauer et al. [8] studied the effects of cooperation and competition on intrinsic motivation and performance, revealing that both attitudes have positive aspects; Peng and co-authors [9] have examined the extent to which motivation plays a role when it comes to cooperative or competitive games. Diverse studies devoted to the analysis of evolutionary games and cooperation on networks [12] have been reported, for instance the introduction of coevolutionary rules to evolutionary games [13]; the analysis of evolutionary dynamics on structured populations [14]; the role of topological properties for the resolution of social dilemmas [15, 16] and, very recently, the cooperation dynamics has been considered within the context of multilayer [17–19] and other networks [5].

Previous models of the dynamics of conflicts have been mainly based on qualitatively defined reaction functions between the actors [20]. Recently, attempts to model cooperative and competitive behaviors in conflicts have considered non-linear differential equations. Liebovitch et al. [21] proposed a two-actor model based on the Gottman et al. [22] mathematical model of the interaction between married couples and formulated appropriate equations and parameters to operationalize the concepts of the cooperative or competitive behavior of actors as described by Deutsch [4, 6]. A later study [23] based on that model considered time delays in the interactions between the actors. Using a local stability analysis together with numerical simulations that study found important dynamic characteristics similar to those observed in real conflict situations. These studies modeled only the interactions between two actors. As noted above, many studies have highlighted the importance of considering many actors and the topology of how they are connected together.

As pointed out by Nowak [24], the structured topology, which defines who interacts with whom, plays an important role, specially in the context of evolutionary game models such as the prisoner’s dilemma [24]. For instance, scale-free topology together with a high clustering coefficient (which characterizes the local structure) seems to be beneficial for the evolution of cooperative structures [25]. Since the pioneering work of Watts et al. [26], many studies have reported the importance of the network topology in systems ranging from genetics to social sciences [25, 27–30], and recently, remarkable universal features present in many different complex networks have been reported [31].

The purpose of this paper is to present the dynamic consequences of the cooperative and competitive behavior of actors interacting in a network. In order to do this, we generalize the two-actor model of Liebovitch et al. to *N*-actors located in a small-world network. In this network there are local interactions between nearest-neighbors but there are also extra connections which form interactions between much more distant actors in the network. We use numerical simulations to compute the social dynamics, namely how the values of the actors change in time. We determine how the distribution of final states of the actors and different local and global measures of the distances between the values of the actors depend on the number of cooperative and competitive actors and the connectivity of those actors in the network. Interestingly, we found that the divergence between the values of the actors is minimized and there is the best trade-off between local and global measures, when there are similar numbers of cooperative and competitive actors.

## N-actors model

We generalize the two-actor conflict model proposed in Ref. [21] to *N*-actors where the actors are represented by the nodes in a network. The activity *x*_*i*_(*t*) which represents the emotional affect of each actor *i* is given by,

$$\begin{array}{c} \frac{dx_{i}\left(t\right)}{dt}=-m_{i}x_{i}\left(t\right)+\sum_{j=1}^{N}a_{ij}c_{j}\tanh x_{j}\left(t\right), \end{array}$$ (1)

where the first term on the right-hand side of this equation represents the auto-dynamic dependence of *x*_*i*_, with *m*_*i*_ the relaxation coefficient or inertia to neutral state. The second term considers the interaction of actor *i* with its neighborhood, according to the connectivity specified by the adjacency matrix *a*_*ij*_. The value *c*_*j*_ represents the influence strength of the *j* ^*th*^-actor, and the nonlinear interaction is modulated through the hyperbolic tangent function. The pairwise interactions are then governed by the sign of *c*_*j*_ and by the behavior of the hyperbolic relationship (see Refs. [21] and [23] for details of the two-dimensional model). When *c*_*j*_ > 0 the effect of *x*_*j*_ on *x*_*i*_ has the same sign as *x*_*j*_, which we therefore identify as a cooperative interaction, while when *c*_*j*_ < 0 the effect of *x*_*j*_ on *x*_*i*_ has the opposite sign as *x*_*j*_, which we therefore identify as a competitive interaction. For convenience, we consider the case *m*_*k*_ = *m* > 0 and ∣*c*_*k*_∣ = *c*, for *k* = 1, …, *N*. A *strong interaction* (or *strong feedback* [21]) is attained if *m* < *c*.

**Fig 1:**
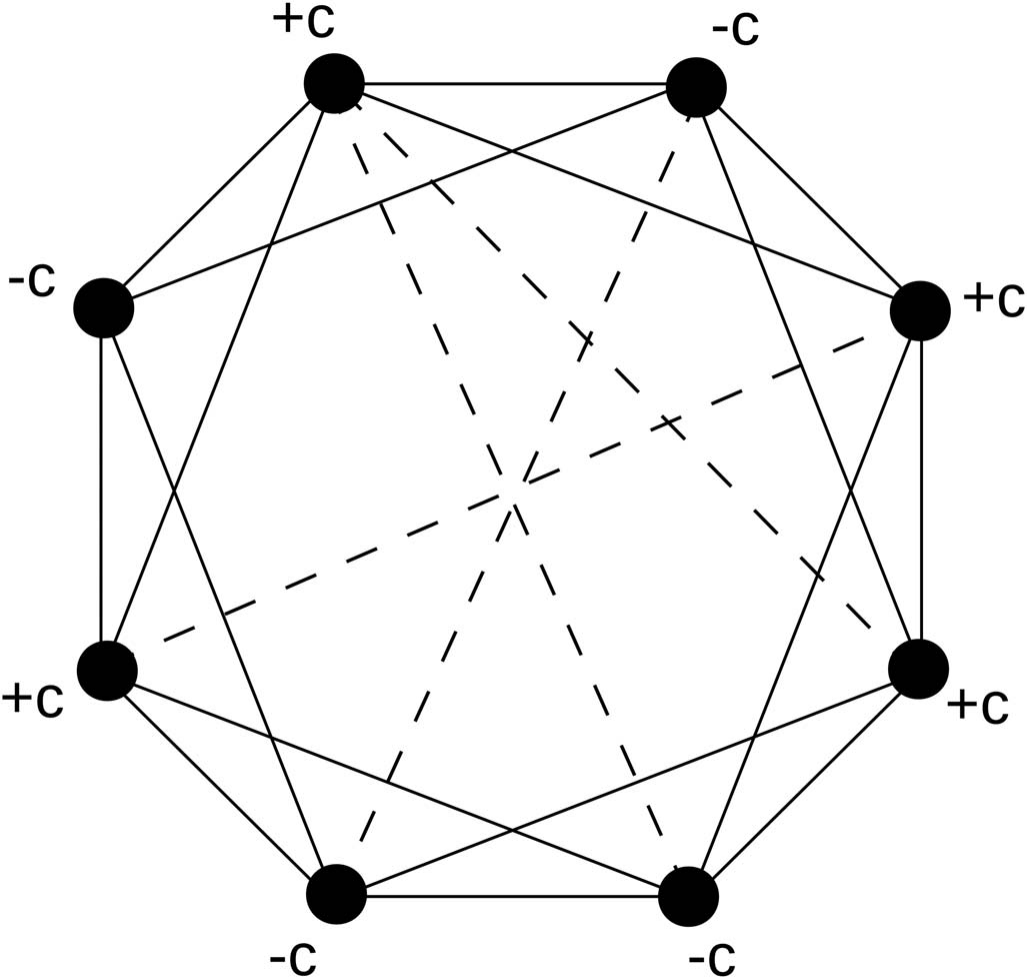
Actors network example. Here labels +*c* and −*c* correspond to cooperative and competitive actor, respectively. In this case, we represent cases for which $r∼\frac{4}{8}=0.5$ and *p* ∼ 0.25. Solid lines correspond to links in the regular ring, while the dashed ones are the extra-connections.

Each actor interacts with the other actors through the connections of the network. The topology of the network is therefore important in determining the dynamics of the system. To generate a Newman-Watts-Strogatz (NWS) small-world network, shown in Fig 1, we start with a regular ring where each actor is connected to two of its nearest neighbors on either side and then we connect pairs of randomly selected nodes with a certain probability, *p*. Note that this topology is slightly different from the original Watts-Strogatz model [26, 32] where long-range connections were re-wired from local connections. In our case, no edges are rewired and *Nk*_0_ *p* is the mean excess connectivity, where *N* is the number of units and *k*_0_ is the number of neighbors in the original network. This NWS network is parametrized by the over-wiring probability *p*, which permits us to generate networks that exhibit the small-world property for intermediate values of *p* [32].

The number of cooperative actors in the network is *N*_+*c*_ and the number of competitive actors is *N*_−*c*_. An important parameter is the *r*, which is the fraction of cooperators of the total number of actors, namely, $r=\frac{N_{+c}}{N}$, where *N* = *N*_+*c*_ + *N*_−*c*_ the total number of actors. If *r* = 0 all actors are competitive, and if *r* = 1 all actors are cooperative.

## Results and Discussion

We use numerical simulations to determine the effects of: (i) the network topology as characterized by the parameter *p* and (ii) the fraction of cooperative actors *r* on the evolution and the final steady-states of the system. Base on our previous studies of two-actor models [21, 23] we chose the values of *m* = 2 for the relaxation coefficient and ∣*c*_*j*_∣ = 3 for the influence strength of the *j* ^*th*^-actor. The initial values of *x*_*i*_ were chosen from a uniform random distribution over [-1, 1]. Next, we evolve the values of *x*_*i*_ in over 50 × 10^3^ time steps time using a 4th order Ruge-Kutta integration method. We save all the values of *x*_*i*_ of all the actors in order to characterize the dynamics and the final steady-state of the system.

**Fig 2:**
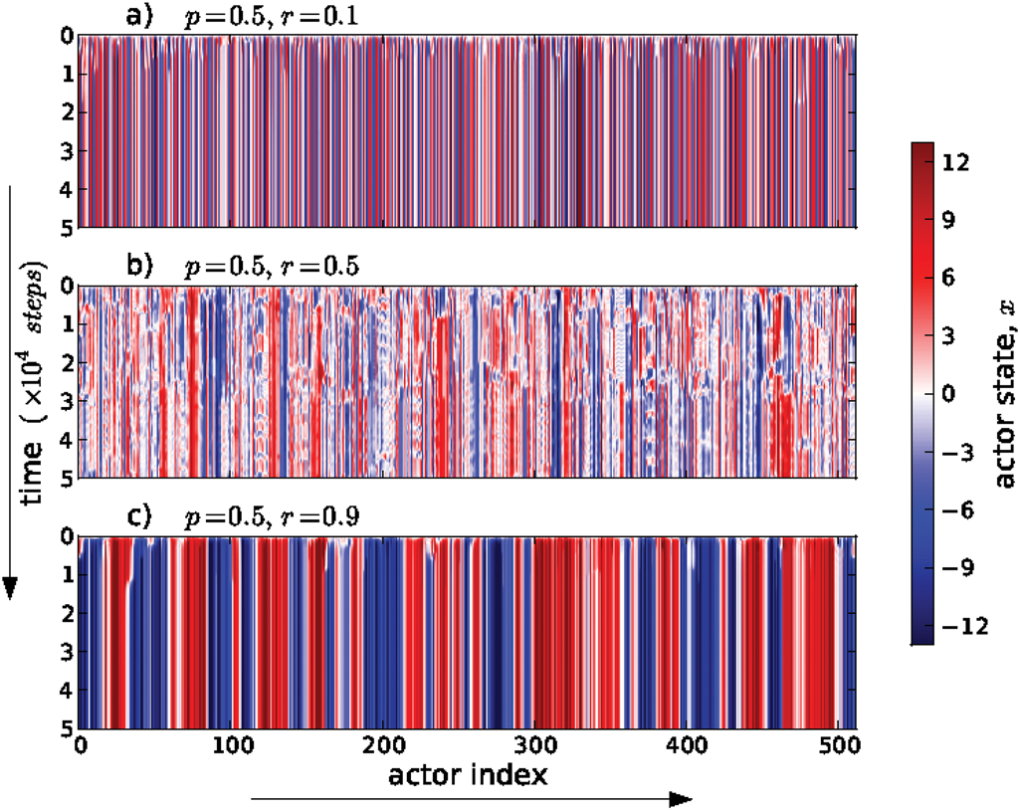
Time evolution of actor’s states for different configurations. Our simulations consider actors placed initially in a regular network with *k*_∘_ nearest neighbor connections. New extra-connections are added with probability *p*. The number of cooperators or competitors are controlled by means of the parameter *r*, which is defined as the ratio of cooperators over the total number of actors. In all cases showed here, we set the over-wiring probability *p* = 0.5, and the cooperativity parameter (a) *r* = 0.1, (b) *r* = 0.5 and (c) *r* = 0.9. The horizontal axis is the index of each actor ordered sequentially so that each actor is adjacent to their nearest local neighbors. Notable differences are observed between the evolutions for different values of the cooperativity ratio (see text for details).

Fig 2 shows representative time evolutions of the system with 512 actors for the over-wiring probability *p* = 0.5 with different values of the fraction of cooperators $r=\frac{N_{+c}}{N}$ with *r* = 0.1, *r* = 0.5 and *r* = 0.9. In these three cases, we started the simulation with the same initial conditions and used the same seed to generate random numbers. For r = 0.1, when most actors are competitive, the system evolves toward a configuration where the neighboring actors tend to be in stable but opposite states, which is why the vertical bands in the top panel in Fig 2 are so narrow, and there are only a few unstable clusters. For *r* = 0.9, when most actors are cooperative, there are also stable clusters, although ones with a larger number of neighbors in the same state, and only a few unstable clusters. These results are consistent with the expectation that predominant competitive behavior will produce smaller regions of similar values compared to predominant cooperative behavior. For *r* = 0.5, when the number of actors that are cooperative matches the number that are competitive, many clusters remain unstable. This may be understood as a reflection of the dynamics of pair-wise interactions. We previously showed that in a two-actor cooperative-competitive interaction the values of the actors oscillates and then reaches a neutral value [21] which may then be more easily de-stabilized in this network model.

**Fig 3:**
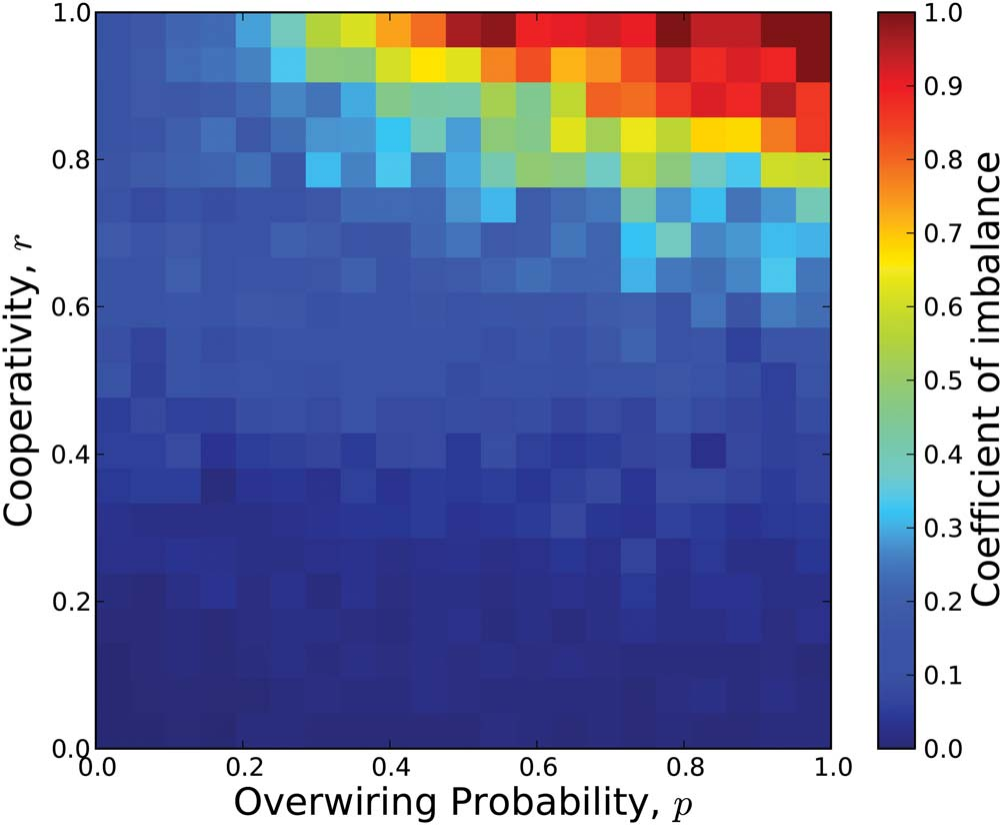
Coefficient of imbalance (CI) for several configurations of conflict actor networks. The phase space of the over-wiring probability *p* vs. the cooperativity ratio *r* is depicted. For low values of *r*, the final steady-states are close to the value *ϕ* ≈ 0, i.e., the averaged states of positive actors is almost equal to the negative ones or viceversa. The value of CI is represented according to the color bar.

Next, we analyze the statistical properties when the system has reached a final steady state. To do this, first we define the average values of the positive (*x* ^+^) and negative (*x* ^−^) states,

$$\begin{array}{c} {\bar{x}}^{+}=\frac{1}{N^{+}}\sum_{i=1}^{N}|x_{i}^{+}|,\;\;{\bar{x}}^{-}=\frac{1}{N^{-}}\sum_{i=1}^{N}|x_{i}^{-}|, \end{array}$$ (2)

where the number of actors for which the final state is positive is *N* ^+^ and for which the final state is negative is *N* ^−^ and *N* = *N* ^+^ + *N* ^−^. We define the coefficient of imbalance, *ϕ*, as

$$\begin{array}{c} \phi =\frac{|{\bar{x}}^{+}-{\bar{x}}^{-}|}{{\bar{x}}^{+}+{\bar{x}}^{-}}, \end{array}$$ (3)

in which the numerator represents the mean distance between the average values of the actors that are positive or negative normalized by the denominator of the sum of those two values. When the final state of the system consists of a symmetric distribution with respect to 0 of the values *x*_*i*_ of the actors, then *ϕ* = 0 and when the values of all the actors are positive or when the values of all the actors are negative, then *ϕ* = 1. Fig 3 shows the results of the average values of *ϕ* for 441 pairs of values of *p* and the fraction of cooperators *r*. Note that for *r* = 0, when all the actors are competitive, the final states of the actors are nearly symmetric around 0 and *ϕ* ≈ 0, for all values of *p*. As *r*, the fraction of cooperative actors, increases *ϕ*, the coefficient of imbalance, increases slightly, indicating a greater degree of imbalance between the number of actors with negative and positive values. However, for high values of *p* and *r*, then *ϕ* is close to 1 because the long distance connections link distant cooperative actors together causing their values to be all positive or all negative.

**Fig 4:**
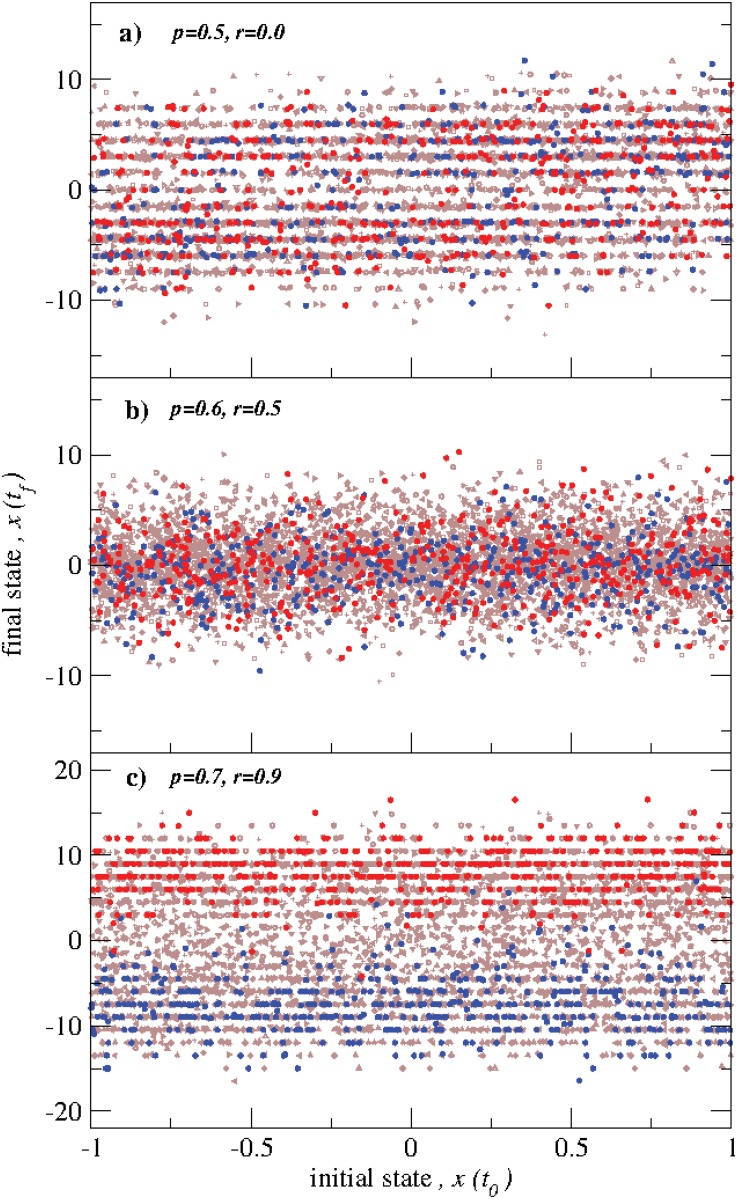
Scatter plots of states *x*(*t*_*f*_) vs. *x*(*t*_*i*_) for different values of *p* and *r*. Here we show the results of 10 independent evolutions, and we have colored two of them in red and blue, in order to show a single realization. (a) For the case *p* = 0.5 and the cooperativity ratio *r* = 0.0 (i.e. when all actors are competitors), the final states are independent of the initial ones and distributed along strips. (b) As in (a) but for *p* = 0.6 and *r* = 0.5 (i.e. the numbers of cooperators and cooperators are equal), for this case the final values tend to be more confined and the strips are not well defined. (c) As in (a) but for *p* = 0.7 and *r* = 0.9, now the strips are present and it is noteworthy that most of the actors tend to have final values either positive or negative.

**Fig 5:**
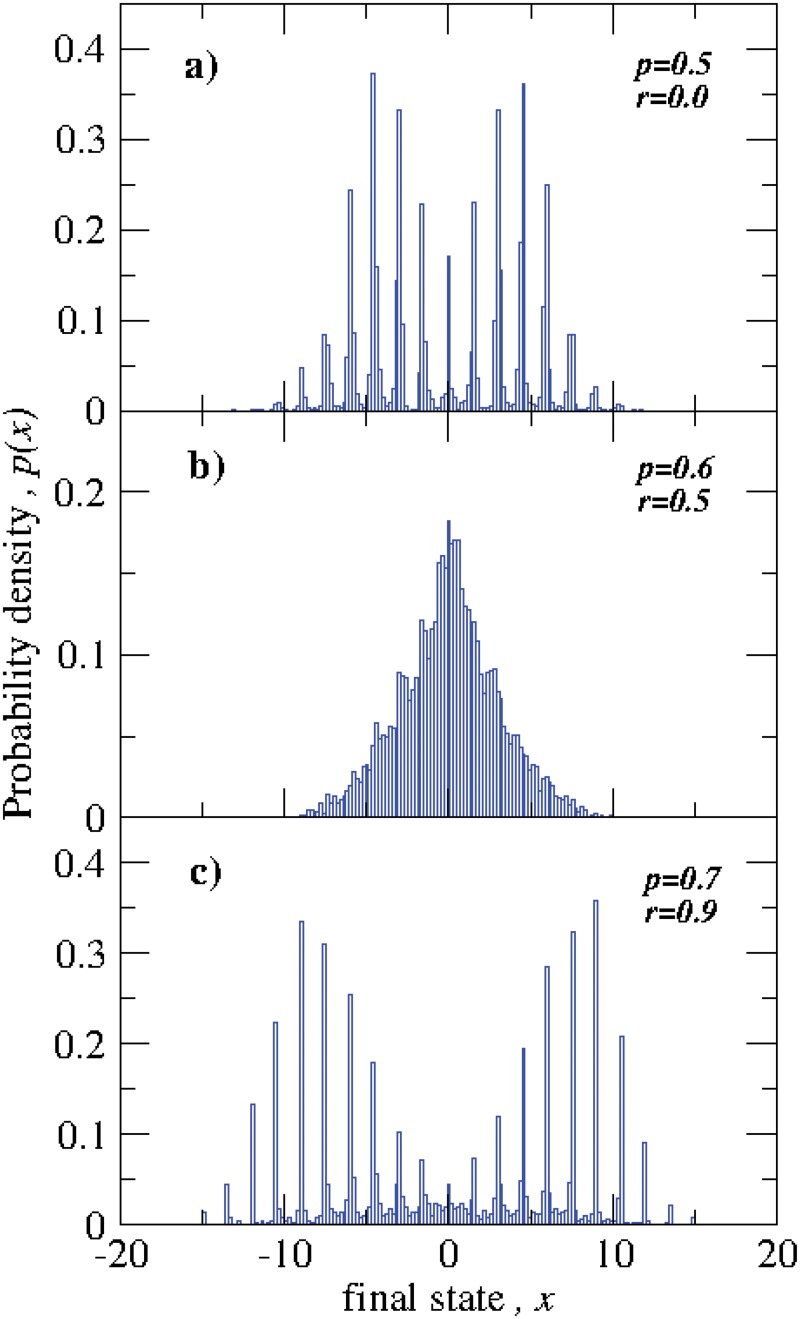
Probability distributions of final states for different values of *p* and *r*. We have constructed the probability density by using the results of 10 independent realizations. For small (*r* = 0.0) and large values (*r* = 0.9) (i.e., when number competitors or cooperators dominates the system, respectively) and intermediate values of *p*, the probability functions of final states exhibit multiple narrowly peaked local distributions, while for the case of equal number of cooperators and competitors, *p*(*x*) is narrow.

Now, we study the effect of the actor’s initial condition on their final state for different values of *p* and *r*. As shown in Fig 4, the final states (*x*(*t*_*f*_)) are independent of their initial values (*x*(*t*_0_)). Most interesting in Fig 4 is the wider amplitude displayed for the low and high values of *r* compared to *r* = 0.5. Moreover the final states for the low and high values of *r* are distributed in strips which are not present when *r* = 0.5. To further explore the distribution of final states, we construct the probability density function, *p*(*x*), of final states, *x*(*t*_*f*_)), for several independent realizations which are shown in (Fig 5). We find that *p*(*x*) exhibit different aspects for different values of *r*. Similar to that found in Fig 4, the distribution *p*(*x*) is overall considerably broader for *r* = 0.1 and *r* = 0.9 with multiple narrowly peaked local distributions with highly populated final states compared to the overall narrower and smoother dispersion found when *r* = 0.5. The results found in Figs 4 and 5, for small and large values of *r*, when either most actors are cooperative or competitive, can be understood from the behaviors of the cooperative-cooperative and competitive-competitive two-actor model [21]. In the network model here, either pair-wise cooperative or pair-wise competitive interactions build up values sequentially from the plateaus of the hyperbolic tangent interaction function. On other hand, when *r* = 0.5, the values of the cooperative-competitive two-actor model oscillate and then reach a neutral value [21], so the values of the actors in the network model here are more likely to remain closer together. Note that since Fig 5 represents the results from 10 independent realizations, some which have more actors in positive final states and some of which have more actors in negative final states, the overall means of *x* for all three cases are near zero.

### Local and Global Distances

We also use global and local metrics to provide important information about the system in terms of the final steady state values of the actors. To do this, we define the mean global distance (*δ*_*G*_) as,

$$\begin{array}{c} \delta_{G}\left(p,r\right)={\left\langle \frac{2}{N(N-1)}\sum_{i>j=1}^{N}|x_{i}-x_{j}|\right\rangle }_{s} \end{array}$$ (4)

where the brackets indicate the average over *s* independent realizations. To consider local conditions, we introduce two local measures: (i) the local average distance, and (ii) the neighborhood average distance. We define the local average distance as,

$$\begin{array}{c} \delta_{L}\left(p,r\right)={\left\langle \frac{1}{N}\sum_{i=1}^{N}\frac{2}{\left(k_{i}+1\right)k_{i}}\sum_{j,k\in \Delta_{i}}|x_{j}-x_{k}|\right\rangle }_{s}\;, \end{array}$$ (5)

where Δ_*i*_ is the set of nearest-neighbors actors of node *i*, including itself, and *k*_*i*_ is its degree. In a similar way, we define the neighborhood average distance as,

$$\begin{array}{c} \delta_{N}\left(p,r\right)={\left\langle \frac{1}{N}\sum_{i=1}^{N}\frac{2}{k_{i}\left(ki-1\right)}\sum_{j,k\in \Delta_{i}^{{}^{\prime }}}|x_{j}-x_{k}|\right\rangle }_{s}\;, \end{array}$$ (6)

where $\Delta_{i}^{\prime }$ is the set of nearest-neighbors of node *i*, without considering *i*. The main difference between Eqs 5 and 6 is that the former statistic is centered on the actor, while for the latter statistic, the distances between the neighbors of a given actor play an important role. In this way, the neighborhood mean distance is related to the clustering coefficient [32].

**Fig 6:**
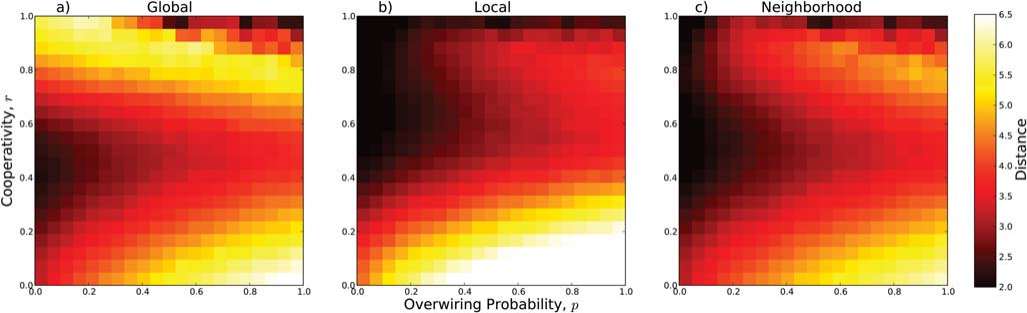
Global and local distances for several configurations of the system. We systematically investigate the behavior of (a) *δ*_*G*_, (b) *δ*_*L*_ and (c) *δ*_*N*_ for different values of the over-wiring probability and the cooperativity actor ratio. (a) For global distance, we observe the smallest value when *p* = 0 and *r* ≈ 0.5, then the distance increases as *r* increases or decreases. The effect of *p* is also to increase the average distance, except for the region of *r* ≈ 1 and *p* ≈ 1, where distances are smaller. (b), (c) For local distances, *δ*_*L*_ increases with the number of extra-connections; while it decreases with the number of cooperators. A similar profile is observed for *δ*_*N*_, except for the fact that large distance values are achieved for *r* ≈ 0 and *p* > 0. Interestingly, for intermediate values of *p*, that is, when the small-world topology is present, and *r* ≈ 0.5, the local and global average distances exhibit small values.

Fig 6, shows the values of these measures for 441 pairs of values of *p* and *r*. The mean global distance in the values *x*_*i*_ of the actors is shown in Fig 6a. For *r* = 0, when all actors are competitive, *δ*_*G*_ increases as *p* increases. Interestingly, as the fraction of cooperators, *r*, increases, the global separation between actors decreases down to a minimum (around *r* ≈ 0.5 and *p* = 0); and then increases, except for a region corresponding to *p* > 0.5 and *r* ≈ 1. We note that this latter region (*p* > 0.5 and *r* ≈ 1), which is also shown in Figs 2c and 4c, is where the coefficient of imbalance is at its maximum, *ϕ* ≈ 1.

The local distance measures in the values *x*_*i*_ of the actors are shown in Figs 6b and 6c. Overall, both *δ*_*L*_ and *δ*_*N*_ increase with increasing *p* because those added long distance links create more local disparities in the values of the actors. Overall, they both also decrease with increasing *r* because increasing cooperation reduces the difference in the values of the actors in a local neighborhood. However, both measures also display shallow local minima in the regions along *r* = 0.5 and *r* = 1.

**Fig 7:**
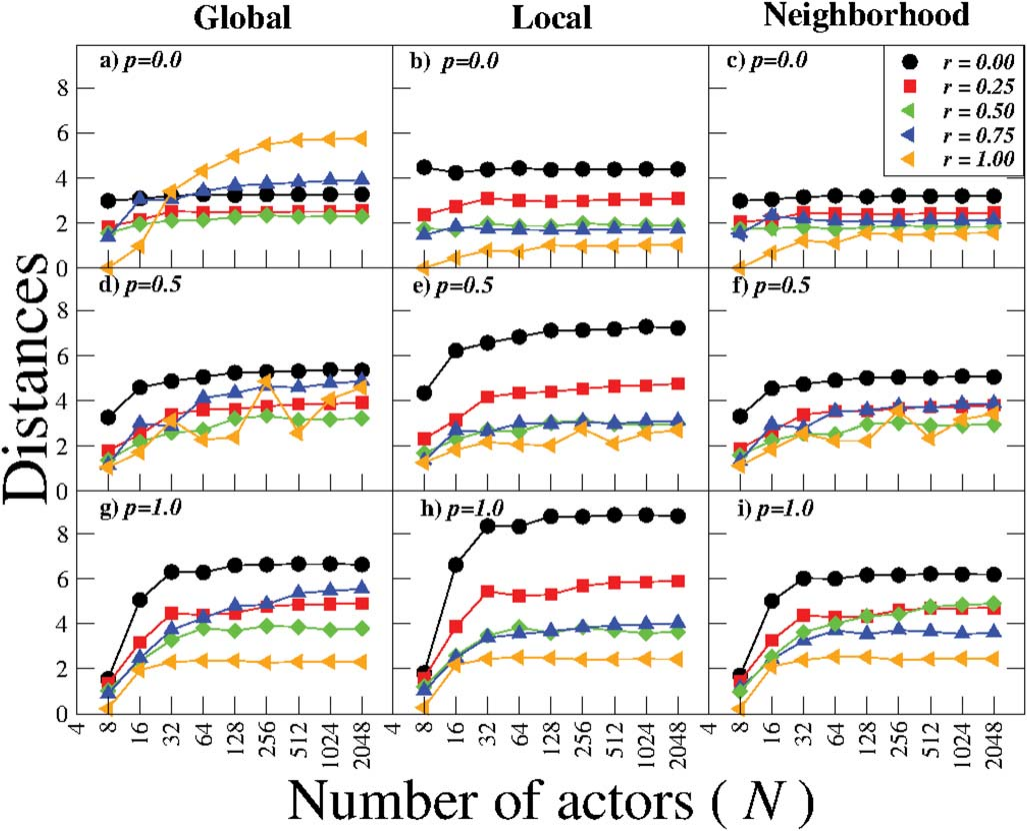
Global and local distances as a function of the system size. Dependence of *δ*_*G*_, *δ*_*L*_ and *δ*_*N*_ on *N* for systems with (a), (b), (c) *p* = 0.0; (d), (e), (f) *p* = 0.5; (g), (h), (i) *p* = 1.0 and different values of *r*. For very small systems local and global distances display a slowly growth, whereas for large systems the distances are independent of the system sizes.

Next, we examine the impact of the system size, *N*, on these local and global distance measures. The dependence of *δ*_*G*_, *δ*_*L*_ and *δ*_*N*_ on the number of actors *N* is shown Fig 7 for several values of *p* and *r*. We find that *δ*_*G*_, *δ*_*L*_ and *δ*_*N*_ all grow slowly for very small system sizes but then saturate as *N* → ∞, indicating that all large systems, *N* > 256, have similar properties (constant value).

Our simulations of different fractions of cooperative actors, *r*, on small-world networks of different fractions of long distance links, *p*, have illustrated interesting behavior in the time evolution and the steady values, *x*_*i*_ of the actors in the networks. We have been able to characterize the final steady states achieved in terms of the probability distributions of the values *x*_*i*_, the coefficient in imbalance between positive and negative values, *ϕ*, and global and local measures of the distance of the values between the actors, *δ*_*G*_, *δ*_*L*_ and *δ*_*N*_. Perhaps our single most salient and interesting result is the finding that for intermediate values of the over-wiring probability, *p*, small-world networks with approximately equal numbers of cooperative and competitive actors, have final steady states with the narrowest distribution of the values of the actors and the smallest values of both the global and local distances between the values of the actors.

## Concluding remarks

We presented the results of a numerical simulation of a non-linear actor, conflict model in which the actors, which are either cooperative or competitive, interact with each other on a small-world network. Perhaps surprisingly, we found that the maximum divergence in the values of the actors is reached when the number of either cooperative or competitive actors dominates the system. On the other hand, we found that the actors have the most similar values and the fewest extremely different values when: 1) there is a roughly equal mix of actors with cooperative and competitive behaviors and 2) the actors interact mostly with their nearest neighbors and sometimes, but not too often, with distant actors. These findings suggest two interesting new questions.

First, one might think that a system functions best when all its elements behave in a similar way and that therefore a human social system would function best when all its actors are cooperative. However, we found here, that having a mixture of different behaviors actually leads to more coherence in the overall functioning of a system. This suggests that not only cooperation but both cooperation and competition acting together play a positive role in the overall effective global functioning of a human social system. This leads us to ask: Is there is a correlation between the cooperation/competition ratio and the degree of effective function in human social systems? Answering this question may also have implications for the practical resolution of conflicts, where value could be seen in having a mixture of different behaviors rather than in seeking similar behaviors in order to reach a consensus to resolve a conflict.

Second, if systems with such mixtures of behaviors and small-world properties function more coherently and therefore more effectively, does this mean that evolutionary processes will drive the evolution of behaviors to produce an approximate balance between cooperators and competitors as well as select their interactions with each other to form a network with small-world properties?
